# On Certain Forms of Headache

**Published:** 1848

**Authors:** 


### Part 3.—Selections.

### ARTICLE I.

*On certain Forms of Headache.* By Dr. Murphy.

[In an essay read before the South London Medical Society, the author described the following varieties of headache: 1st, Periosteal headache; 2d, Rheumatic headache; 3d, Nervous headache; 4th, Anæmic headache; 5th, Congestive headache.]

1. Periostitis of the cranium is seldom met with unless after a mercurial course, in a scrofulous constitution, and is generally found to invade either the coronal or parietal bones.—The diagnosis of headache arising from this cause, although not difficult, has sometimes been erroneous. The pain is severe, and confined to one or more of the localities above mentioned; it is increased at night in bed, by stimulating drinks, and by pressure. A raised surface can be detected at the site of the pain, and on inquiry it will be found that more than one course of mercury has been given. The treatment should consist in the application of the emplast. hydrargyri spread on thick leather, and the exhibition of the iodide of potassium with morphia and tincture of digitalis.

2. Another form of headache is the rheumatic or fibrous, which is located in the tendon of the occipito-frontalis, the temporal aponeurosis, and tendinous insertions of the muscles at the back of the head. It is usually preceded by rheumatism of other parts, and is increased by muscular movements. Warm coverings, when they can be applied, usually relieve it, also sinapisms; and, if the pain is very severe, leeches and cupping, and a few doses of calomel with opium, seldom fail to give relief. Gout may also attack the same parts, but may be diagnosed by our previous acquaintance with the habits of our patient. In both cases, the pain is intermitting, changes its locality, and is felt to be external, and the health is not affected.

3. The next form belongs to the class of spinal irritation, is very frequent, and met with exclusively in females during the menstrual periods, and attacks mostly the left side of the head; the pain is intermittent, shooting, and lancinating; may be fixed for days, and is most severe at the temple (when it is termed clavus hystericus), and next at the parietal protuberance and occiput: it proceeds from the sub-occipital nerve, and, if the exit of the nerve is pressed upon, pain, more or less severe, is complained of, extending along the whole course, or at certain sites only of the nerve—as at the temple, nape of the neck, parietal protuberance, &c.: it is usually increased during the menstrual period, and is generally a complaint of unmarried females, between the 23d and 35th years of life, and is indubitably a form of hysteria. The menses are usually profuse or difficult, the bladder irritable, and there are ill-defined, painful sensations about the pelvis; and three forms of neuralgia co-exist. The irritation of the sub-occipital nerve must be traced to the ovaries, being only present where these exist, and while capable of fulfilling the function of menstruation; and our treatment must be primarily directed to remove any congestion, or irritation of these peculiar organs; and, secondarily, to lessen the pain of the nerves. The author advises the daily use of hip-baths, or sea-bathing, where possible: attention to prevent accumulation in the rectum; abstinence from stimulants; mental employments; inf. valerian c. digitalis, with pills of assafœtida; occasionally, general or only local bleeding; and when these fail, a gentle mercurial action, the cold bath being during the time omitted. As local means, he recommends belladonna plasters, veratrine ointment, sinapisms, or blistering. When, however, the patient is exhausted by leucorrhœa or profuse menstruation, with symptoms of chronic inflammation of the womb or ovaries, the treatment becomes more doubtful; but the author prefers the trial of a tonic treatment, and advises the exhibition of the valerianate of zinc and quinine as especially efficacious, and the sulphate of iron in infusion of valerian when there are evidences of confirmed chlorosis. This headache may be termed the *nervous* headache; it also assumes another form, which may be termed the *cutaneous* headache, and is the hemicrania of our forefathers: it seems to be located in the integuments of one half—usually the left side—of the head, which is so exquisitely sensible as scarcely to bear the least touch of the finger, and the pain never passes the mesial line.

4. Another form of headache is that arising from deficiency of blood within the cranium, and coming on after hemorrhages, exhausting discharges, or any other debilitating causes: the best examples arise in chlorosis. It is increased by the erect, diminished by the recumbent posture; it is not a very painful form, but is often attended with impaired vision; its cause may be traced to diminished muscular power of the heart, which palpitates on slight exertion: there are also dyspnœa, pale face, and other symptoms of a feeble circulation, with a sinking pain at the epigastrium, and craving appetite. If the true cause of this headache be mistaken, and depletion used, paralysis has been known to supervene; but if the debility be removed, the muscular power of the heart is easily increased, and the most useful remedies are, steel by itself or combined with quinine, full diet, and the recumbent posture.

5. The last form of headache alluded to by the author arises from excess of blood, and may exist as a passive or congested, or as an active or inflammatory state. The former, arising from various known causes of congestion, is diagnosed by the constant heavy pain at the anterior part of the head, increased by the recumbent posture, sense of chilliness, slow, feeble pulse, tendency to vomiting, and pain in the lumbar region, caused by congestion of the spinal cord. It is a dangerous form of headache, and has, in the depressing diseases, proved fatal in a few hours; but, in other cases, has lasted weeks without much mischief. The treatment should be to induce reaction as soon as possible by the warm bath or an emetic. If the headache persists with hot skin, leeches to the inner nares will be found of value; applied to the temple, they debilitate without relieving the pain in the head, and they are altogether inadmissible when this co-exists with typhus or scarlatina. Blisters may also be applied, and diaphoresis produced by the usual means; cold applications to the head the author considered useless, and even likely to increase the congestion. Care is also requisite that mere congestion should not, by the use of stimuli, be forced into inflammation, which is the next stage, if resolution or fatal termination does not take place. The author regards idiopathic phrenitis as a most rare disease, and hydrocephalus acutus as congestion, not inflammation. Phrenitis is well marked by the tensive pain increased on stooping, by the bright eye, hot skin, nausea and vomiting, tendency to delirium, and occasional twitching of the muscles of the face; the most active antiphlogistic measures should be used.

There were other forms of headache easy of diagnosis, but of these the author would only mention the constant pain of the head in children, with emaciation and want of sleep, and which diagnosed tubercles of the brain. In the headaches of pregnant females, referred to the centre of the head, and attended with a remarkably small pulse, and in which, if bleeding is neglected, convulsions, abortion, and too often death, are apt to supervene; and, lastly, the pain of the head occurring after a night's debauch, the cause of which, whether in the stomach or affected organ, the author considered not to have been sufficiently investigated.—*Med. Gaz.*, in Rank.'s Ab.

